# Management of aromatase inhibitor-associated bone loss (AIBL) in women with hormone-sensitive breast cancer: An updated joint position statement of the IOF, CABS, ECTS, IEG, ESCEO, IMS, and SIOG

**DOI:** 10.1016/j.jbo.2025.100694

**Published:** 2025-06-11

**Authors:** Peyman Hadji, Matty Aapro, Nasser Al-Dagri, Majed Alokail, Emmanuel Biver, Jean-Jacques Body, Maria Luisa Brandi, Janet Brown, Cyrille Confavreux, Bernard Cortet, Matthew Drake, Peter Ebeling, Erik Fink Eriksen, Ghada El-Hajj Fuleihan, Theresa A. Guise, Nick C. Harvey, Andreas Kurth, Bente Langdahl, Willem Lems, Radmila Matijevic, Eugene McCloskey, Rossella Nappi, Santiago Palacios, Georg Pfeiler, Jean-Yves Reginster, René Rizzoli, Daniele Santini, Sansin Tuzun, Catherine Van Poznak, Tobias De Villiers, M. Carola Zillikens, Robert Coleman

**Affiliations:** a Frankfurt Centre for Bone Health and Endocrinology, Frankfurt, Germany; b Philipps University of Marburg, Marburg, Germany; c Cancer Center, Clinique de Genolier, Switzerland; d Biochemistry Department, College of Science, King Saud University, Riyadh, Saudi Arabia; e Division of Bone Diseases Geneva University Hospitals and Faculty of Medicine Rue Gabrielle Perret-Gentil, Geneva, Switzerland; f CHU Brugmann, Department of Medicine, Free University of Brussels, Brussels, Belgium; g Fondazione FIRMO, Florence, Italy; h Division of Endocrinology and Metabolism, McMaster University, Hamilton, Canada; i Division of Clinical Medicine and Weston Park Cancer Centre, University of Sheffield, Sheffield, UK; j University of Lyon, Bone Metastasis Expert Center (CEMOS) Cancer Institute of Hospices Civils de Lyon, Rheumatology Department, Centre Hospitalier Lyon Sud, Pierre Bénite, France; k Centre for Cancer Research Lyon (CRCL) - UMR INSERM 1052 CNRS 5286, University Claude Bernard Lyon 1, Lyon, France; l Department of Rheumatology, CHU Lille and University of Lille, Lille, France; m Metabolic Bone Service, Hospital for Special Surgery, NY, USA; n Department of Medicine, School of Clinical Sciences at Monash Health, Monash University, Clayton, Victoria, Australia; o Pilestredet Park Specialist Centre and Faculty of Odontology, University of Oslo, Oslo, Norway; p Calcium Metabolism and Osteoporosis Program, WHO Collaborating Center for Metabolic Bone Disorders, Department of Medicine, American University of Beirut, Beirut, Lebanon; q Department of Endocrine Neoplasia and Hormone Disorders, The University of Texas MD Anderson Cancer Center, Houston, TX, USA; r MRC Lifecourse Epidemiology Centre and NIHR Southampton Biomedical Research Centre, University of Southampton, Southampton, UK; s University Hospital Southampton NHS Foundation Trust, Southampton, UK; t Orthopedic Institute Dr. Baron and Colleagues, Goethe University, Frankfurt am Main, Frankfurt, Germany; u Department of Endocrinology and Internal Medicine, Aarhus University Hospital, Aarhus, Denmark; v Department of Clinical Medicine, Aarhus University, Aarhus, Denmark; w Department of Rheumatology, Amsterdam UMC, Amsterdam, the Netherlands; x Faculty of Medicine, University of Novi Sad, Novi Sad, Serbia; y Centre for Metabolic Bone Diseases and the Mellanby Centre for Musculoskeletal Research, Division of Clinical Medicine, School of Medicine & Population Health, University of Sheffield, Sheffield, UK; z Department of Clinical, Surgical, Diagnostic and Pediatric Sciences, University of Pavia, Pavia, Italy; aa Research Center for Reproductive Medicine, Gynecological Endocrinology and Menopause, IRCCS S. Matteo Foundation, Pavia, Italy; ab Palacios Clinic for Women’s Health, Madrid, Spain; ac Department of Gynecology and Gynecological Oncology, Medical University of Vienna, Vienna, Austria; ad Geneva University Hospitals and Faculty of Medicine, Geneva, Switzerland; ae Department of Medical Sciences and Biotechnology, University “Sapienza”, UOC Medical Oncology, Rome, Italy; af Faculty of Medicine, Istanbul University-Cerrahpaşa, Istanbul, Turkey; ag Department of Internal Medicine, Division of Hematology and Oncology, University of Michigan, Ann Arbor, MI, USA; ah Mediclinic Panorama and Department of Gynecology, Stellenbosch University, Cape Town, South Africa; ai Department of Internal Medicine, Erasmus MC Bone Center, Erasmus University Medical Center, Rotterdam, the Netherlands; aj Weston Park Hospital and University of Sheffield, Sheffield, UK

**Keywords:** Breast cancer, Osteoporosis, Aromatase Inhibitor, Fracture, Bisphosphonate, Denosumab

## Abstract

**Background:**

Women with hormone-responsive breast cancer who receive adjuvant endocrine treatment with aromatase inhibitors (AI) are known to be at higher fracture risk due to a marked increase in bone resorption. In 2017, several interdisciplinary cancer and bone societies involved in the management of women with AI-associated bone loss (AIBL) published a joint position statement comprising evidence-based recommendations and a practical management algorithm for the assessment of fracture risk and optimal treatment of this patient population.

**Patients and methods:**

In order to provide updated recommendations that reflect recent advances in the assessment and management of AIBL since publication of the 2017 joint position statement, a systematic literature review was undertaken to identify relevant studies for analysis, including systematic reviews and meta-analyses. Individual trials identified were assessed for their level of evidence based on design, size, follow-up, and evaluation of safety, as well as the impact of bone directed treatments on breast cancer outcomes.

**Results:**

New evidence was combined with the existing recommendations to provide an updated joint position statement regarding fracture risk assessment and implementation of bone-directed therapy.

**Conclusion:**

Current published literature, including recent clinical trial reports, systematic reviews and meta-analyses, continue to affirm the high risk of fractures in women with breast cancer who are receiving adjuvant AI treatment, a risk which has been observed to increase with the commonly used approach of extended duration AI therapy (>5 years). Risk factors for fracture and risk assessment in this patient population as well as the most suitable treatment modalities have been updated. Finally, the influence of bone protective treatments on breast cancer outcomes such as incidence of bone metastasis and breast cancer related overall survival have been included.

## Highlights

• Aromatase inhibitors (AI) are the standard treatment for estrogen-responsive breast cancer.
• AI reduce cancer recurrence but also increase bone resorption.
• AI-associated bone loss (AIBL) leads to an increased risk of fracture.
• This update to the 2017 joint position statement on the management of AIBL includes recent data, reviews and meta-analyses.
• An updated evidence-based AIBL treatment algorithm is presented.

## 1 Introduction

Breast cancer is currently the most commonly diagnosed cancer worldwide and the leading cause of cancer mortality in women, making it a significant global public health concern [1,2]. Data from the World Health Organization (WHO) estimate that in 2022, 2.3 million women were diagnosed with breast cancer globally and this resulted in 670,000 deaths [3]. Despite the high observed prevalence of breast cancer, mortality rates from the disease have substantially decreased in many countries over the past few decades due to a combination of increased public awareness, implementation of screening programs to enable earlier diagnosis as well as the availability of improved treatments and individualized management strategies [4,5]. A recent analysis of breast cancer mortality in the USA from 1975 to 2019 using simulation models found a 58 % reduction over that time period [4]. The report suggested that contributors to this overall decrease were improved treatment for stage I to III breast cancer (47 %) and metastatic disease (29 %), as well as the routine use of mammography screening (25 %). However, the positive outcomes observed with the latest breast cancer therapies have meant an increase in the numbers of patients being treated with these effective agents, which has in turn resulted in an increase in long-term treatment-emergent adverse effects that include bone loss, osteoporosis and fractures [6]. While primary disease management is essential to patient survival following a breast cancer diagnosis, loss of bone mineral density (BMD) and the associated increased risk for fragility fractures with some systemic breast cancer therapies need to be addressed as these effects can negatively impact both patient morbidity, mortality and quality of life [[7], [8], [9]].

The majority of breast cancers are hormone responsive, expressing estrogen receptors (ER) and/or progesterone receptors (PR) with around 80 % of all breast cancers categorized as ER-positive. Adjuvant endocrine therapy following resection of the localized tumor, generally administered for 5–10 years (in addition to adjuvant chemotherapy and targeted treatments in certain subgroups), is standard of care to prevent recurrence and improve survival outcomes [10]. These endocrine treatments aim to either directly target ER receptors, thereby preventing estrogen binding and stimulation of tumor growth, or to suppress estrogen production. Tamoxifen, a selective estrogen receptor modulator (SERM), was previously the standard therapy for ER-positive breast cancer in postmenopausal women. However, over the past two decades aromatase inhibitors (AI), in particular the third-generation agents anastrozole, letrozole and exemestane, which prevent the aromatization of endogenous androgens into estrogen, have largely replaced tamoxifen as the treatment of choice for postmenopausal women with ER-positive breast cancer due to their superior effect on cancer recurrence, the development of cancer in the opposite breast, and breast cancer-related mortality as well as having an improved safety profile [11,12]. AI are usually used from the outset of endocrine therapy but in some clinical situations may be initiated following several years of tamoxifen therapy. Endocrine therapy is usually recommended for a minimum of five years with extended endocrine therapy to complete 7–10 years of treatment considered for patients with a relatively poor prognosis and at high risk for recurrence [13]. AI are also increasingly used alongside ovarian suppression therapy in premenopausal women with high-risk, ER-positive breast cancer [14]. Endocrine treatment is usually initiated after adjuvant chemotherapy has been completed so such patients are not suitable for bisphosphonates to prevent disease recurrence but will experience rapid bone loss that may require intervention.

To maintain skeletal homeostasis and optimal BMD, a careful balance between bone resorption and bone formation is required, a process which is regulated by a range of factors, including estrogen. This balance can be disrupted by either the natural reduction in endogenous estrogen that occurs after the menopause or by some breast cancer therapies, resulting in an accelerated rate of bone loss that exceeds bone formation.

### 1.1 Development of an updated joint position statement

Observations of the significant negative impact that AI and other breast cancer therapies can have on bone health and structure in postmenopausal women reinforce the need for clear strategies to routinely assess and appropriately treat this patient group alongside their primary disease management. Our 2017 joint position statement provided a systematic review of the available literature at that time, undertaken by several interdisciplinary cancer and bone societies involved in the management of AI-associated bone loss (AIBL), and proposed an algorithm to help direct healthcare professionals in the assessment and treatment of their postmenopausal breast cancer patients receiving AI therapy [10]. This article provides an update to that position paper incorporating information on advances in assessment of fracture risk and the latest clinical data on optimal treatment strategies with antiresorptive agents.

## 2 Methods

### 2.1 Literature review

Searches were undertaken of PubMed® and MEDLINE® (National Library of Medicine, Bethesda, MD), as well as other databases, to identify clinical trials, systematic reviews and meta-analyses regarding antiresorptive bone-targeted agents used for the prevention and treatment of AIBL from 2016 to 2024. In addition, the Cochrane Register of Controlled Trials and databases of ongoing and unpublished trials https://www.clinicaltrials.gov were searched. Additional information was obtained from abstracts presented at international meetings including the St. Gallen Breast Cancer Conference, European Breast Cancer Conference (EBCC), San Antonio Breast Cancer Symposium (SABCS), and American Society of Clinical Oncology (ASCO) annual meetings and breast cancer symposia.

An evidence-based medicine approach was used to determine when to initiate antiresorptive therapy for AIBL, to determine the appropriate choice and duration of antiresorptive therapy and define follow-up/monitoring procedures. All new reports published since the 2017 joint position statement were reviewed and the available data assessed for the level of evidence used to guide treatment recommendations, as previously described [10].

## 3 Results

### 3.1 The impact of breast cancer therapy-associated bone loss on fracture risk

Bone loss due to breast cancer therapies has been widely reported and is associated with various different treatment modalities [6,10,15]. In contrast to the case in premenopausal women, tamoxifen has not been found to have a negative impact on BMD or fracture risk in postmenopausal women [16]. Indeed, results of a systematic review and meta-analysis suggest it may potentially preserve bone mass in postmenopausal women [17]. AI, which are now more commonly prescribed due to improved anticancer efficacy, are associated with a higher fracture risk than tamoxifen due to their ability to almost completely suppress circulating and tissue estrogen levels, and are known to accelerate bone loss and increase fracture risk in this population beyond that seen with natural menopause [15,[17], [18], [19], [20], [21]].

The risk of fracture in women is based on a range of underlying modifiable and non-modifiable risk factors, including a family history of osteoporosis, poor nutrition (in particular a lack of calcium and vitamin D), physical inactivity, active smoking and heavy alcohol consumption, low body mass index, chronic corticosteroid treatment (>6 months) and, importantly, age with an increased risk observed post- menopause or with early menopause (<45 years of age) [[22], [23], [24], [25]]. Data from the WHO estimate that the overall global prevalence of osteoporosis is 19.7 %, although this varies substantially between countries and regions [23]. In the female population, it is estimated that one in three women over the age of 50 years will sustain a fragility fracture resulting in increased morbidity and mortality, particularly if these occur at the spine or hip [26].

In women undergoing breast cancer treatment, the use of agents known to accelerate bone loss need to be integrated into the estimation of fracture risk [24]. A population-based study of 5,146 younger breast cancer patients (aged 20–39 years) in Taiwan evaluated fracture risk following treatment with either AI, radiotherapy or monoclonal antibodies [27]. A multivariate proportional hazards analysis showed that all these treatment regimens were significantly associated with a high risk of fracture with patients who received AIs for more than 180 days at a particularly high risk with a hazard ratio (HR) for fracture of 1.77.

Studies have shown that AIBL in postmenopausal women with hormone-sensitive breast cancer, where estrogen is already naturally depleted, can lead to a 2–4 fold increase in bone loss compared to the usual postmenopausal decrease in BMD, leaving them at high risk of fragility fractures [10]. A meta-analysis of 30 randomized controlled trials (RCTs) including 117,974 breast cancer patients found a significantly higher incidence of osteoporotic fractures, in AI users versus those not treated with AI (controls), particularly for vertebral fractures [28]. The relative risk (RR) for hip fractures and non-vertebral fractures was 1.18 (p < 0.001 in each case) versus controls, while the RR for vertebral fractures was 1.84 (p < 0.001).

Additionally, fat body mass (FBM) may be associated with fragility-related fractures in patients with breast cancer who undergo aromatase inhibitor therapy. In a single-center, cross-sectional study of 556 postmenopausal women with early-stage breast cancer receiving AI treatment the proportion of vertebral fractures in the aromatase inhibitor-treated group was 20.0 % in patients with low FBM versus 33.3 % in patients with high FBM (p = 0.04). If these data are confirmed, obesity could be included in the algorithm for assessing fracture risk and selecting patients to receive bone resorption inhibitors [29].

The increasing use of extended duration of AI treatment (up to 10 years) adds to this risk. A systematic review and meta-analysis of seven trials of 16,349 breast cancer patients treated with either extended-duration AI, placebo or no treatment found that longer treatment with AI was associated with a significantly higher risk of fractures (odds ratio [OR]: 1.34; p < 0.001) [30]. A later meta-analysis of eight RCTs including 15,966 patients found that while a longer duration of AIs therapy for postmenopausal patients with early breast cancer could further improve DFS compared with standard adjuvant AI therapy, this longer exposure to AI was also associated with an increased relative risk of low energy fractures (RR = 1.59; p = 0.002) and osteoporosis (RR = 1.53; p = 0.005) [31].

Bone loss has also been observed in women receiving chemotherapy (CT) for breast cancer. In younger women this is largely due to the induction of menopause but adverse effects on bone have been observed also in older postmenopausal women. A small, prospective, single-arm observational study of 18 non-osteoporotic, postmenopausal women with breast cancer undergoing chemotherapy treatment (complemented with glucocorticoids to reduce treatment related side effects) evaluated changes in bone microstructure and volumetric measured using high-resolution peripheral quantitative computed tomography (HRpQCT) as well as BMD determined by dual-energy X-ray absorptiometry (DXA) of the distal radius and distal tibia [8]. HRpQCT measurements 6 months post-chemotherapy indicated a significant decrease in median total volumetric BMD (distal tibia − 4.5 % and distal radius − 2.3 %), cortical volumetric BMD (−1.9 % and − 0.8 %, respectively), and trabecular volumetric BMD (−1.1 % and − 3.0 %, respectively). BMD reductions were most marked at weight-bearing sites, namely the distal tibia, lumbar spine and femur.

### 3.2 Assessment of fracture risk in women with breast cancer

Accurate assessment of fracture risk is key to evaluating the need for intervention with antiresorptive therapies. A retrospective analysis of a cohort of 130 women with breast cancer undergoing AI treatment found that preventive assessment of bone health and timely intervention with therapy at the start of AI treatment allows the identification of patients at high fracture risk and may contribute to preventing bone events in these patients [32].

As outlined in our 2017 position paper, the most widely used technique for the assessment of osteoporosis and fracture risk in women with breast cancer is measurement of BMD T-scores using DXA, typically at the spine and hip [10]. However, when monitoring treatment effect on BMD, there can sometimes be discordance between spine and hip measurements, mainly due to degenerative changes in the spine. This phenomenon was evaluated in a population-based BMD Registry in Canada in 6,093 women aged ≥ 40 years who were undergoing treatment for osteoporosis [33]. Subjects were followed up for a mean of 12.1 years and the results showed that the total hip site provided a better indicator of an anti-fracture effect than measurements of the lumbar spine. A cross-sectional study explored the prevalence and determinants of vertebral fractures in 263 postmenopausal women with ER-positive early breast cancer before and during AI therapy using DXA to assess BMD and a quantitative morphometric approach to identify vertebral fractures [34]. The analysis found that AI therapy was associated with a high prevalence of radiological vertebral fractures (31.2 % in AI −treated versus 18.9 % in AI-naïve subjects; odds ratio 1.90; p = 0.03) that was shown to be independent of BMD values during the adjuvant AI treatment, suggesting the need for multiple approaches to the assessment of fracture risk in this population.

BMD measurement alone has been supplemented in recent years with the use of the FRAX® algorithm (https://frax.shef.ac.uk/FRAX), a validated online tool developed by the University of Sheffield in the UK, for calculating an individual’s 10-year probability of developing a hip fracture or a major osteoporotic fracture (MOF) with or without BMD data [35]. The FRAX® tool was developed for use in the general population and was not specifically designed for use in patients with breast cancer undergoing AI treatment. When using the FRAX® tool, AI exposure is not a parameter that can be inputted directly but it has been proposed to categorize it as ‘secondary osteoporosis’ to reflect the associated increased risk of fracture.

Analysis of data from a Canadian population‐based registry that included women aged ≥ 40 years initiating AI for breast cancer who had at least 12 months’ AI exposure (n = 1,775), women with breast cancer not receiving AI (n = 1016), and women from the general population (n = 34,205) found that fracture risk estimated without BMD and using AI use coded as ‘secondary osteoporosis’ significantly overestimated 10‐year risk [36]. In contrast, when BMD was included in the fracture probability calculation, there was no significant difference between observed and predicted fracture risk. However, FRAX® was able to stratify the risk of MOF, hip fracture specifically and any fracture equally well in all subgroups.

A study undertaken in Denmark of 116 women with early breast cancer about to start AI assessed whether the inclusion of BMD data impacted FRAX® risk calculation [37]. The authors concluded that DXA scanning should be performed to provide BMD data for inclusion in FRAX® to avoid overestimation of fracture risk before AI treatment commenced.

In recent years, new measurement techniques have emerged to allow more detailed insights into bone health and microarchitecture. Trabecular bone score (TBS) utilizes grey-level texture measurements on lumbar spine DXA images to capture information relating to trabecular microarchitecture and has been shown to be an independent indicator of increased fracture risk [38,39]. A study of 100 patients with early-stage ER-positive breast cancer treated with AI assessed elevated fracture risk using BMD alone, BMD plus FRAX® and a combination of BMD, FRAX® and TBS [40]. The use of multiple assessment techniques incrementally improved the identification of patients at increased fracture risk with the combination of all three procedures maximizing the number detected. Following AI treatment, changes in TBS were independent of changes in BMD. These results align with the findings of an earlier retrospective cohort study of 34 women treated with AI with a mean follow-up of 2.1 years which found that the decrease in TBS following AI treatment was significantly smaller than that of lumbar BMD (−2.1 versus −5.9 %; p = 0.002) [41].

HRpQCT is a three-dimensional imaging technique that enables discrimination of trabecular and cortical bone compartments, providing densitometric and structural information. Previously, HRpQCT was used predominantly in the research setting, however it is now showing considerable promise in clinical practice use to improve fracture prediction when used alongside DXA BMD measurements and assessment of clinical risk factors [[42], [43], [44]]. This was demonstrated in the observational study described earlier of women undergoing chemotherapy where HRpQCT was able to provide additional granularity to the assessment of bone health in this population [8]. However further studies are needed to confirm any incremental benefits of this technique.

### 3.3 Anticancer benefits of adjuvant bisphosphonates

In recent years, bisphosphonates have been recognized as having a range of potential extra-skeletal effects, including anti-tumor, immunomodulatory, anti-inflammatory and anti-diabetic properties [[45], [46], [47], [48], [49], [50]]. A systematic review and meta-analysis of 34 studies that evaluated the association between bisphosphonate use and the occurrence of any type of cancer suggested that treatment with these agents may have an additional protective effects against the development of colorectal, breast and endometrial cancer outside of their effect on bone resorption [51].

In postmenopausal women with breast cancer experiencing AIBL, in addition to the positive effects on bone health, bisphosphonate treatment has been reported to have both direct and indirect anticancer benefits, reducing skeletal metastases and improving rates of both recurrence free and overall survival [10,25,52]. These findings are reflected in this updated joint position statement and treatment algorithm for AIBL management.

### 3.4 Evidence and recommendations for selection of antiresorptive therapies for the prevention of AIBL

Recent clinical data and technical advances in fracture risk assessment have been evaluated and incorporated into a comprehensive range of national and international guidelines outlining the criteria for antiresorptive use in women with breast cancer. The most recent of these, as identified in our literature search for each of the key professional bodies or national organizations, are summarized in Table 1 [10,12,15,[53], [54], [55]]. Our literature search also identified five meta-analyses published from 2015 onwards summarizing the results of studies of antiresorptive agents that include data on the prevention of AIBL in postmenopausal women with breast cancer (Table 2) [[56], [57], [58], [59], [60]]. The overarching message from these reports is that the monoclonal antibody denosumab, as well as both intravenous and oral bisphosphonates, can effectively prevent AIBL in patients with breast cancer. However, each individual compound has its own efficacy and tolerability profile and supporting evidence base of clinical trials and real-world studies, as discussed below. Evidence from clinical trials is also supported by the results of real-world clinical practice studies [61]. It should be noted however that the complete range of available antiresorptive agents are not licensed in all countries and specific regulatory approval for their use in early breast cancer is lacking.

**Table 1:** Summary of recent guidelines for antiresorptive use in women with breast cancer.

Source	Suitable patients	Antiresorptive agent	Dose	Duration of treatment/follow-up
Hadji P, et al. 2017 [10] Previous joint position statement of the IOF, CABS, ECTS, IEG, ESCEO, IMS, and SIOG.	All women receiving AI therapy with a T-score < − 2.0 or with a T-score of < –1.5 SD with one additional risk factor, or with ≥ 2 risk factors (excluding BMD)	Zoledronate (recommended when effects on disease recurrence are the priority)	4 mg i.v. q6 months	For the duration of AI therapy
		Denosumab (recommended when fracture risk is the dominant concern).	60 mg s.c. q6 months	

Bouvard B, et al. 2019 [53] French recommendations	All women receiving AI therapy who have a history of severe osteoporotic fracture and/or a T-score value < –2.5. FRAX score should be used to guide treatment decisions in patients whose T-score is between − 1 and − 2.5	Risedronate	35 mg PO/week	Follow-up at 2 years to reassess whether treatment should be continued
		Alendronate	70 mg PO/week	
		Zoledronate (not licensed in France for this population)	4 mg i.v. q6 months or 5 mg i.v./year	
		Denosumab (only as second-line treatment; not licensed in France for this population)	60 mg s.c. q6 months	

Shapiro CL, et al. 2019 [54] ASCO Clinical Practice Guidelines	Postmenopausal women receiving AI therapy who have osteoporosis (T scores of ≤ 2.5 in the femoral neck, total hip, or lumbar spine) or those who are at increased risk of osteoporotic fractures based on clinical assessment or risk assessment tools (10-year probability of 20 % for major osteoporotic fractures or 3 % for hip fractures based on the US-adapted FRAX tool)		Doses as indicated for osteoporosis	When DXA scans show T-scores have improved, discontinuation of the antiresorptive agent can be considered
		Alendronate	10 mg PO/day or 70 mg/week	
		Risedronate	5 mg PO/day or 35 mg PO/week	
		Ibandronate	2.5 mg PO/day or 150 mg PO/month	
		Zoledronate	5 mg i.v./2 years	
		Denosumab	60 mg s.c. q6 months	

Coleman R, et al. 2020 [12] ESMO Clinical Practice Guidelines	Postmenopausal women receiving AI therapy for > 6 months with either a BMD T-score of < –2 or with ≥ 2 risk factors for fracture or annual bone loss on treatment is confirmed to exceed 5 %	Denosumab as first-line therapy Alendronate Risedronate Ibandronate Zoledronate	60 mg s.c. q6 months 70 mg PO/week 35 mg PO/week 150 mg PO/month 4 mg i.v. q6 months	For the duration of AI therapy (up to 5 years); monitor BMD every 2 years to reassess

Waqas K, et al. 2021 [15] Updated guidance on management of CTIBL in women with early-stage breast cancer	All women receiving AI therapy with a BMD T-score < –2.0 SD or with ≥ 2 clinical risk factors including a BMD T-score < –1.0 SD	Denosumab as first-line therapy Zoledronate Ibandronate Clodronate	60 mg s.c. q6 months 4 mg i.v. q6 months 150 mg PO/month 1,600 mg PO/day	Repeat DXA scans at 2 years to reassess whether treatment should be continued

Eisen A, et al. 2022 [55] ASCO-OH (CCO) guideline update	All postmenopausal women (natural or therapy-induced) with primary breast cancer should be considered for adjuvant BP therapy. Factors influencing the decision to recommend treatment include risk of recurrence, risk of side effects, financial toxicity, drug availability, patient preferences, comorbidities, and life expectancy	Start BP therapy early (within 3 months of definitive surgery or within 2 months of completion of adjuvant chemotherapy Clodronate Ibandronate Zoledronate	1,600 mg/day 50 mg PO/day 4 mg i.v. q6 months (for 3 years) or 4 mg i.v. q3 months (for 2 years)	2–3 years

Professional Societies: ASCO, American Society of Clinical Oncology; ASCO-OH (CCO), American Society of Clinical Oncology–Ontario Health (Cancer Care Ontario); CABS, Cancer and Bone Society; ECTS, European Calcified Tissue Society; ESCEO, European Society for Clinical and Economics Aspects of Osteoporosis, Osteoarthritis and Musculoskeletal Diseases; ESMO, European Society for Medical Oncology; IEG, International Expert Group for AIBL; IMS, International Menopause Society; IOF, International Osteoporosis Foundation; SIOG, International Society for Geriatric Oncology.

Other abbreviations: AI, aromatase inhibitor; ASCO, American Society of Clinical Oncology; BMD, bone mineral density; BMI, body mass index; BP, bisphosphonate; DXA, dual energy X-ray absorptiometry; FRAX, fracture risk assessment tool; GnRH, gonadotropin-releasing hormone; i.v., intravenous; LS, lumbar spine; NCCN, National Comprehensive Cancer Network; PO, orally; q, every; s.c., subcutaneous; SD, standard deviation; TH, total hip; UK, United Kingdom.

**Table 2:** Meta-analyses of studies of antiresorptive agents for prevention of AIBL in postmenopausal women with breast cancer.

Study description and antiresorptive agent(s) evaluated	Patient population	Impact on BMD		Fracture risk
		Lumbar spine	Total hip	
Early Breast Cancer Trialists’ Collaborative Group, 2015 [56] Meta-analysis of 26 RCTs of BP versus control (open label or placebo) Median 5.6 years follow-up • Zoledronate • Ibandronate • Pamidronate • Risedronate • Alendronate • Clodronate	18,766 women in total; 18,206 [97 %] in trials of 2–5 years of BP; 11,767 postmenopausal	Not reported	Not reported	Bone fractures were reduced versus control (RR 0·85, 95 % CI 0·75–0·97; p = 0·02).

Mei M, et al. 2020 [57] Meta-analysis of 13 RCTs • Zoledronate • 1 year follow-up	7,375 women receiving adjuvant therapy for early breast cancer; 8/13 studies reported data for postmenopausal women (n = 4,915)	Significant improvement in LS BMD. MD between ZA treated and non-ZA groups: 0.06 g/cm^2^ (95 % CI: 0.05–0.07, p < 0.00001)	Significant improvement in TH BMD. MD between ZA treated and non-ZA groups: 0.04 g/cm^2^ (95 % CI: 0.03–0.04, p < 0.00001)	Not reported

Miyashita H, et al. 2020 [58] Meta-analysis of 16 RCTs • Risedronate • Zoledronate • Denosumab • 1- and 2-years follow-up	7,699 women receiving AI treatment following surgery for breast cancer: Risedronate (n = 312) Zoledronate (n = 1,708), Denosumab (n = 1,838) No upfront treatment (n = 3,841) Pre- or postmenopausal status not reported but median age 49–65 years	All agents significantly increased LS BMD at 1 and 2 years versus no upfront treatment; ZA and DM resulted in significantly higher LS ΔBMD (5.45 % and 5.64 % at 1 year, and 7.26 % and 7.97 % at 2 years, respectively) than RI	All agents significantly increased TH BMD at 1 and 2 years versus no treatment; ZA and DM resulted in significantly higher TH ΔBMD (3.34 % and 4.65 % at 1 year, and 3.75 % and 5.31 % at 2 years, respectively) than RI	DM and RI reduced the incidence of fracture significantly versus no treatment (RR 0.51 [0.38–0.67] and RR 0.54 [0.35–0.83], respectively). DM associated with lower fracture risk than BP (RR 0.60 [0.38–0.94]).

Bassatne A, et al. 2022 [59] Meta-analysis of 14 RCTs • Zoledronate (7) • Oral BP: risedronate (4), ibandronate (2) • Denosumab (1) • Up to 3 years follow-up	7,231 postmenopausal women receiving AI treatment for early breast cancer	ZA: MD in LS BMD 5.4 % versus controls at 1 year and 7.3 % versus delayed treatment at 2 years Oral BP: MD in LS BMD 3.4 % versus controls at 1 year and 4.2 % at 2 years DM: MD in LS BMD 6 % versus controls at 1 year and 8 % at 2 years	ZA: MD in TH BMD 3.6 % versus controls at 1 year and 4.7 % versus delayed treatment at 2 years Oral BP: MD in TH BMD 2.1 % versus controls at 1 year and 3.3 % at 2 years DM: MD in TH BMD 4 % versus controls at 1 year and 6 % at 2 years	ZA: Non-significant decrease of 30 % (RR 0.7 [0.3–1.4]) in morphometric vertebral fracture risk at 3 years versus delayed treatment Oral BP: Some studies report numerical decrease in fracture incidence but results equivocal DM: Reduction in fracture incidence of 50 % compared to placebo

Adams A, et al. 2024 [60] Cochrane Database Review; network meta-analysis of 34 RCTs • Risedronate • Zoledronate • Alendronate • Ibandronate • Pamidronate • Clodronate • Denosumab	33,793 women with early and locally advanced breast cancer BMD data from 9 trials (n = 1,166) Fracture risk data from 16 trials (n = 19,492)	Estimated BMD with no treatment: total T-score −1.34 IB: T-score −0.77; MD 0.57 (95 % CI −0.05–1.19); slight increase versus no treatment/placebo ZA: T-score −0.45; MD 0.89 (95 % CI 0.62–1.16); slight increase versus no treatment/placebo RI: T-score −1.08; MD 0.26 (95 % CI −0.32–0.84); little to no difference versus no treatment/placebo AL: T-score 2.36; MD 3.70 (95 % CI −2.01–9.41); results uncertain.	Not reported*	Estimated 70/1,000 women with no treatment/placebo had fractures Decrease in fractures versus no treatment/placebo observed with: CL: 42/1,000; RR 0.60 (95 % CI 0.39–0.92) IB: 40/1,000; RR 0.57 (95 % CI 0.38–0.86) RI: 39/1,000; RR 0.56 (95 % CI 0.15–2.16) Slight decrease observed with: ZA: 55/1,000; RR 0.79 (95 % CI 0.56–1.11) DM: 51/1000; RR 0.73 (95 % CI 0.52–1.01) PA: Increases fracture risk

Abbreviations: AI, aromatase inhibitor; AIBL, aromatase inhibitor-associated bone loss; AL, alendronate; BMD, bone mineral density; ΔBMD, change in BMD from baseline; BP, bisphosphonates; CI, confidence interval; CL, clodronate; DM, denosumab; IB, ibandronate; LS, lumbar spine; MD, mean difference; PA, pamidronate; RCT, randomized controlled trial; RI, risedronate; RR, relative risk; TH, total hip; ZA, zoledronate. *In this analysis, data were extracted for the most common site, 'lumbar spine' only, with the most common unit, 'T-score', measured with dual-energy X-ray absorptiometry to collect data which would be comparable in a quantitative analysis.

#### 3.4.1 Denosumab – Level of evidence i

Denosumab is a human monoclonal antibody that inhibits bone resorption by neutralizing RANKL, a key mediator of osteoclast formation, function, and survival. It is administered subcutaneously, typically at a dose of 60 mg every 6 months for the duration of AI therapy. The 2017 joint position statement which analyzed the results of major trials of denosumab for the prevention of AIBL assigned it as having Level I evidence in this indication and recommended its use when fracture risk (rather than disease recurrence) was the dominant concern [10]. Our current analysis of the latest published treatment guidelines (Table 1) supports this conclusion.

Denosumab is recommended as a first-line therapy for AIBL prevention by the European Society for Medical Oncology (ESMO) [12] and in the 2021 updated guidance on management of cancer treatment-induced bone loss (CTIBL) [15]. It is also recommended as a treatment option in the ASCO Clinical Practice Guidelines [54]. In the French national guidelines, denosumab is only suggested as a second-line option in the French national guidelines because it is not licensed in France for this indication [53].

Miyashita and colleagues evaluated the results of 16 studies of 7,699 women receiving AI treatment following surgery for breast cancer (Table 2), 1,708 of whom received denosumab [58]. Denosumab, and also the intravenous bisphosphonate zoledronate, were associated with greater increases in BMD at both the lumbar spine and total hip than the oral BP, risedronate. Denosumab was also associated with a lower risk of fracture than either of the bisphosphonates. In their analysis of 14 RCTs, which included one study on denosumab, Bassatne and colleagues reported a reduction in fracture incidence of 50 % with denosumab versus placebo [59]. They also found a mean difference in lumbar spine BMD of 6 % versus controls at 1 year and 8 % at 2 years, with corresponding figures for total hip BMD of 4 % versus controls at 1 year and 6 % at 2 years. These values were numerically higher than those reported in studies of intravenous zoledronate or oral bisphosphonates. A recent study investigating 12 months of denosumab treatment versus placebo in 68 premenopausal women with ER-positive early-stage breast cancer receiving gonadotropin-releasing hormone analogues and AI reported sustained BMD and HRpQCT values compared to a significant decrease with placebo [62].

A 2024 Cochrane Database Review and network meta-analysis of 34 RCTs of antiresorptive agents suggest that bisphosphonates (excluding alendronate and pamidronate) or denosumab compared to no treatment or placebo likely results in increased BMD and reduced fracture rates. Overall fracture rate was estimated to be 70 per 1000 participants with no treatment/placebo. Treatment with clodronate (42 fractures per 1000; RR 0.60, 95 % CI 0.39 to 0.92) or ibandronate (40 fractures per 1000; RR 0.57, 95 % CI 0.38 to 0.86) decreased the number of fractures compared to no treatment/placebo (high certainty). Denosumab (51 fractures per 1000; RR 0.73, 95 % CI 0.52 to 1.01), zoledronic acid (55 fractures per 1000; RR 0.79, 95 % CI 0.56 to 1.11) and oral risedronate (39 fractures per 1000; RR 0.56, 95 % CI 0.15 to 2.16) probably slightly decrease the number of fractures; compared to no treatment/placebo [60].

#### 3.4.2 Intravenous bisphosphonates – Level of evidence I

The 2017 joint position statement assigned intravenous bisphosphonates as having Level II evidence for the prevention of AIBL. Subsequent publication of the results of the AZURE study [63] and the conclusions of other reviews and meta-analyses [56,60], suggest that the evidence for intravenous bisphosphonates should now be designated as Level I. The AZURE study was a multicenter, randomized phase III trial evaluating the addition of zoledronate 4 mg to standard therapy (neo/adjuvant chemotherapy and/or endocrine therapy) for 5 years in 3,360 pre- and post-menopausal patients with stage II/III early breast cancer. Adjuvant zoledronate was found to significantly reduce the incidence of fractures in this patient cohort, with a 5-year fracture rate of 3.8 % compared to 5.9 % in the control arm. The addition of zoledronate also significantly increased the time to first fracture (HR: 0.69; p = 0.0053). Notably, the majority of the fracture prevention benefit occurred after disease recurrence (HR: 0.30; p < 0.001) with relatively little effect on fracture risk in women with a sustained remission of their underlying cancer. With regard to the long-term duration of treatment effect, a recent analysis from the AZURE study reported a persistent suppression of bone resorption for as long as five years after session of zoledronate treatment [64].

From a clinical practice perspective, intravenous bisphosphonates have several advantages compared to oral formulations, namely that they are easy to administer at routine clinic visits and are not associated with compliance issues or concerns about absorption. Compared to denosumab, using intravenous bisphosphonates avoids any rebound effect when treatment is stopped and obviates the necessity for sequential treatment. Bisphosphonates are also relatively inexpensive and universally available with generally mild to moderate, easily manageable adverse effects, mainly the acute phase response with the initial dose. This can largely be avoided by a concomitant 3-day glucocorticoid treatment [65]. In addition, less common acute adverse events include a reduction in renal function (risk decreases with adequate hydration and reduces infusion rates), hypocalcemia that can largely be prevented by ensuring adequate vitamin D levels before treatment is initiated and ocular side effects (iritis/uveitis).

#### 3.4.3 Oral bisphosphonates – Level of evidence II

The 2017 joint position statement assigned oral bisphosphonates as having Level III–IV evidence for the prevention of AIBL, however analysis of recent data and conclusions from subsequent reviews and meta-analyses, particularly for clodronate, ibandronate and risedronate, suggests that the level of evidence for these compounds should now be designated as II [56,60].

The majority of the data supporting the use of oral bisphosphonates are derived from small-scale studies not designed to show a fracture risk reduction, so confirmation from studies using larger data sets is needed. In contrast to intravenous bisphosphonates, oral formulations have variable gastro-intestinal absorption and need to be taken on an empty stomach. They are also associated with a relatively high rate of gastrointestinal adverse effects [66]. Importantly, oral bisphosphonates have been reported to be associated with poor levels of persistence with and adherence to treatment, which can negatively impact treatment efficacy [67,68]. In common with intravenous formulations, they are relatively cheap and universally available.

Discontinuation of denosumab in women with postmenopausal osteoporosis is known to be associated with a ‘rebound phenomenon’ whereby the cessation of therapy is associated with a rapid increase in markers of bone turnover, loss of BMD and an increased risk of fractures, in particular multiple vertebral fractures, to that seen prior to denosumab treatment [[69], [70], [71]]. The exact cellular mechanisms underlying this phenomenon remain to be determined but it is thought to involve the osteoclast recycling pathway [[72], [73], [74]].

Considering this phenomenon, it is recommended that on cessation of denosumab treatment, an alternative antiresorptive agent should be commenced within 6 months after the last denosumab injection in order to mitigate against post-treatment BMD loss. The currently available data suggest that preferably intravenous infusion of zoledronate or an oral bisphosphonate administered for at least one year should be used. This should be accompanied by measurement of bone turnover markers after 3 months of treatment to confirm suppression of bone turnover and annual BMD monitoring [69].

Medication-related osteonecrosis of the jaw (MRONJ) is a relatively rare event in the context of the dosing schedules of bisphosphonates and denosumab used for the prevention of bone loss, however, reports of its incidence vary. A review of 467,654 women receiving treatment for osteoporosis in the United Kingdom Clinical Practice Research Datalink [75] showed the absolute risks are low (∼ 0.05 % after 5 years and ∼ 0.18 % after 10 years) and elevated risks diminished to near zero within 6 to 9 months of discontinuation. In the breast cancer setting, it has been suggested that the risk on MRONJ is generally < 1 % in studies that use a relatively low dose of antiresorptive agents, and in a range of trials of the use of bisphosphonates in breast cancer patients without metastases, the reported incidence ranged between 0.3 % and 1.2 % [76] In the AZURE trial, the incidence of confirmed MRONJ was 2.1 % (26 of 33 suspected cases).The risk of MRONJ can be minimized by a careful dental management and checking before treatment initiation, so the benefits of antiresorptive therapy almost always outweigh the risks of the potential development of this uncommon complication of treatment [77,78]. Healthcare teams need to be aware of the possibility of MRONJ and educate patients on the importance of good dental hygiene as a successful preventative strategy. If invasive dental procedures are needed, the use of prophylactic local and systemic antibiotics are recommended [79]. In addition, even less common adverse events include atypical fracture at around 1 per 10,000 person years on treatment [80]. Fear of this adverse event has inappropriately reduced patient adherence to treatment for osteoporosis with benefits again hugely outweighing the tiny risk of experiencing this event.

### 3.5 Treatment and follow-up recommendations

**Fig. 1:**
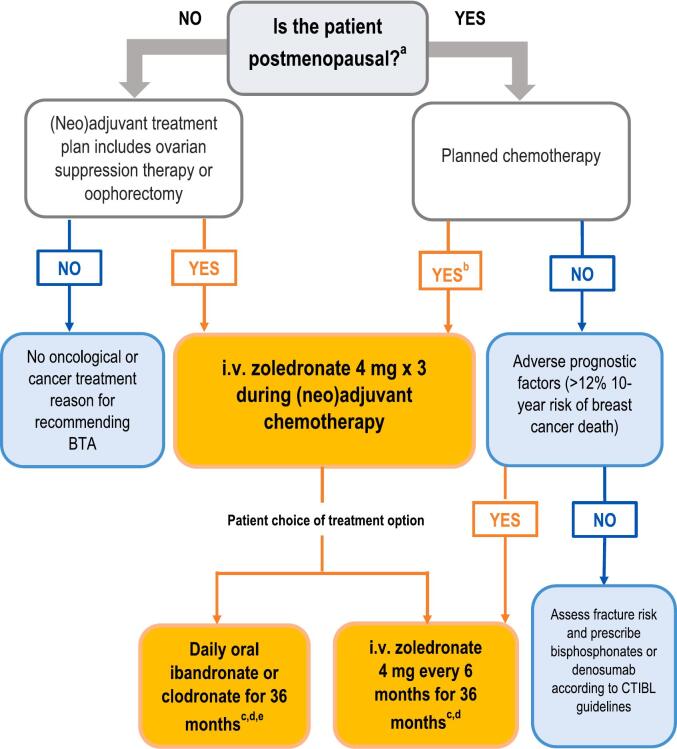
Recommended algorithm for use of bone-targeted treatments in early breast cancer. Adapted from Coleman et al, 2020 [12]. ^a^ If not clinically assessable (i.e. hysterectomy/IUD) then ensure age > 55 and/or serum FSH is in postmenopausal range (patient must not be receiving concurrent therapies that can affect the hypophyseal–pituitary–gonadal axis). ^b^ Patients already on weekly oral bisphosphonates for osteoporosis should be considered for a treatment change and follow algorithm. ^c^ Include vitamin D3 800–2000 IU (plus calcium 1000 mg daily if low calcium diet). ^d^ May switch from oral to i.v. therapy or vice versa if tolerability issues. ^e^ Daily oral ibandronate (50 mg/day) or clodronate (1,600 mg/day) as in the SWOG S0307 trial [85]. Duration of treatment is not well defined and may vary between 2 and 5 years. Abbreviations: BTA, bone-targeted agent; CTIBL, cancer treatment-induced bone loss; i.v., intravenous; IUD, intrauterine device; FSH, follicle-stimulating hormone.

The selection of bone-targeted treatment regimen for the prevention of AIBL in breast cancer patients will depend on their age, menopausal status and breast cancer treatment plan (Fig. 1). In this algorithm postmenopausal women (or premenopausal women commencing ovarian suppression therapy or bilateral oophorectomy as (neo)adjuvant treatment) who are at intermediate to high risk of recurrence should be considered for bisphosphonate treatment alongside other adjuvant systemic treatments including AI to reduce the risks of breast cancer recurrence and death as well as protect against bone loss. For the 50–60 % of women at relatively low risk for recurrence, bone loss and subsequent fracture risk is perhaps of greater concern and fracture risk should be assessed and bisphosphonate or denosumab therapy commenced as necessary according to these and other CTIBL guidelines [15].

### 3.6 Fracture prevention

**Fig. 2:**
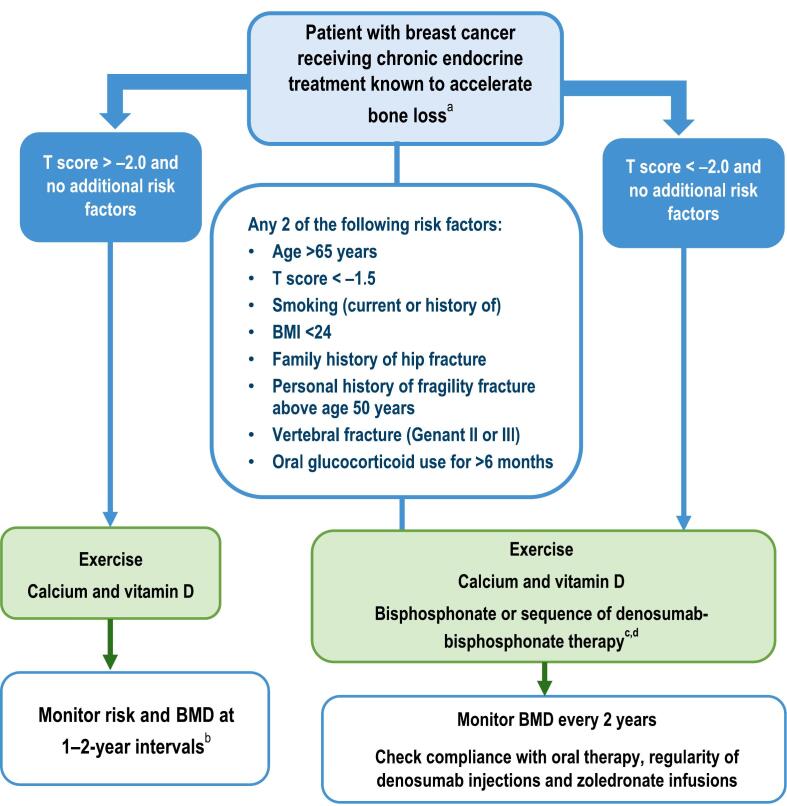
Recommended algorithm for managing bone health in women receiving aromatase inhibitor therapy for breast cancer. Adapted from Hadji et al, 2017 [10] and Coleman et al, 2020 [12]. ^a^ Includes AIs and ovarian suppression therapy/oophorectomy. ^b^ If patients experience an annual decrease in BMD of ≥ 10 % at any site (or ≥ 4–5 % in patients who were osteopenic at baseline) using the same DXA absorptiometry machine, secondary causes of bone loss such as vitamin D deficiency should be evaluated and antiresorptive therapy initiated. Use the lowest T-score from spine or hip. ^c^ Either denosumab (60 mg every 6 months) or six-monthly intravenous zoledronate (4 mg) as first-line treatment (for the duration of endocrine treatment/together typically for up to 5 years). Discontinuation of denosumab should be followed up by an appropriate antiresorptive. ^d^ Weekly oral alendronate (70 mg) or risedronate (35 mg) or monthly oral ibandronate (150 mg) for the duration of endocrine treatment/typically for up to 5 years. Abbreviations: AI, aromatase inhibitor; BMD, bone mineral density; BMI, body mass index.

Based on the wealth of evidence from meta-analyses of RCTs and the distillation of this evidence into national and international guidelines, a recommended algorithm for managing bone health in women receiving AI therapy for breast cancer is proposed (Fig. 2). Recent evidence confirms the algorithm proposed in the 2017 joint position statement, but this has been updated to align with recent international guidelines. In women with a T score > –2.0 and no additional risk factors, the focus should be on ensuring they are having sufficient physical exercise (the World Health Organization has recently published guidelines on what constitutes adequate physical activity in adults [81]) and have an adequate intake of vitamin D and calcium, with risk and BMD monitored at 1–2 yearly intervals. In those with any two of a range of risk factors (see Fig. 2) or those with a T score < –2.0 and no additional risk factors, denosumab or bisphosphonate therapy should be commenced, along with the same guidance on exercise and vitamin D/calcium intake. BMD should then be monitored every two years. When an oral bisphosphonate is prescribed it is critical to reinforce to patients the importance of compliance with therapy to ensure the best possible outcomes. As detailed in the 2017 joint position statement, in the setting of osteoporosis, due to the stringent dosing requirements for oral bisphosphonates, persistence with long term therapy has frequently reported to be suboptimal which can have a negative impact on fracture rates [10].

To help inform treatment selection, a comparison of antiresorptive agents in term of their administration and dosing, as well as their advantages and limitations, are summarized in Table 3.

**Table 3:** Comparison of antiresorptive agents. []

Antiresorptive agent	Dose*	Advantages	Limitations	Long-term safety/efficacy
Oral bisphosphonates (second generation)		• A simple, self-administration regimen	• Limited efficacy data available • Need to follow strict dosing guidelines • High potential for poor treatment adherence and persistence • No data for risedronate or alendronate to assess effects on underlying breast cancer	• Established in the osteoporosis but not AIBL setting • Generally well tolerated • Adverse events are rare and generally mild and manageable (e.g., ONJ, AFF)
Alendronate	70 mg PO/week (or 10 mg PO/day)			
Ibandronate	150 mg PO/month			
Risedronate	35 mg PO/week			

Intravenous bisphosphonate Zoledronate	4 mg q6 months	• Efficacy data from large trials with long follow-up • Can be administered during routine twice yearly oncologist visits • Compliance can be ensured	• Intravenous administration must be undertaken by a healthcare provider	• Established in the osteoporosis and AIBL settings • Generally well tolerated • AEs are rare and generally mild and manageable (e.g., ONJ, AFF)

Subcutaneous denosumab	60 mg q6 months	• Can be administered during routine twice yearly oncologist visits or by a healthcare provider • Compliance can be ensured	• Rebound effect after treatment cessation: sequence denosumab-bisphosphonate required • Relatively high cost	• Established in the osteoporosis and AIBL settings • Anti-fracture efficacy also established in both settings • Generally well tolerated • Adverse events are rare and generally mild and manageable (e.g., ONJ, AFF)

Abbreviations: AEs, adverse events; AFF: atypical femur fracture; AIBL, aromatase inhibitor-associated bone loss; ONJ, osteonecrosis of the jaw; q, every.

* Note that higher doses are generally given in the setting of oncology compared with those given for prevention and treatment of osteoporosis.

The optimum duration of bone protective treatment in the setting of AI use remains uncertain in the context of extended use beyond five years. Most clinical guidelines suggest that bisphosphonates should be used for 3 to 5 years in patients at high risk for fractures, as this period provides significant benefit in reducing fracture risk. During this time, bisphosphonates effectively increase BMD and reduce the incidence of fractures [82]. After an initial treatment period of 3–5 years, extending bisphosphonate therapy beyond 5 years may not provide substantial additional benefit in fracture prevention but can increase the risk of side effects [82]. Additionally, studies have shown that the benefits of bisphosphonate treatment may be sustained even after discontinuation for a period, suggesting that long-term use is not always necessary to maintain skeletal health [83].

Data on the duration of effects on BMD and bone turnover markers from the AZURE trial with zoledronate indicate that efficacy is maintained for at least five years after treatment discontinuation [64]. Patients were randomized to receive 19 doses of zoledronate 4 mg over a 5-year period or a control group and BMD and bone turnover markers assessed after treatment was completed and for a further 5 years of follow-up. As expected, mean BMD, T-scores and Z-scores were higher in the zoledronate treated patients. Bone turnover markers were significantly lower than those in the control arm (α- and β-urinary C-telopeptide of type-I collagen, serum intact pro-collagen I N-propeptide all p < 0.00001 and serum tartrate-resistant acid phosphatase 5b, p = 0.0001). The differences in BMD persisted at all assessed skeletal sites throughout the 5-year follow-up period. Some offset of bone turnover inhibition occurred in the 12 months following completion of zoledronate treatment but thereafter throughout the 5 years of follow-up, all bone markers remained suppressed in the zoledronate treated patients compared to the control patients. These results suggest BMD monitoring and further use of bone targeted therapy to prevent AIBL during extended therapy would seem unnecessary.

### 3.7 Disease recurrence prevention – Adjuvant bisphosphonates

More than 35 randomized clinical trials investigating the use of adjuvant bisphosphonates in early breast cancer have been performed with varying results. However, overall it can be concluded that treatment with these agents reduces bone metastases and breast-cancer deaths in postmenopausal women, over and above their effects on bone health [84]. The importance of this significant accompanying survival benefit of adjuvant bisphosphonate use in this patient population has led to their recommendation in treatment guidelines for bone health in cancer issued by the key professional bodies in oncology, ESMO [12] and ASCO [55].

The optimum bisphosphonate and schedule and duration of therapy to prevent disease recurrence have not been fully defined. Trials comparing agents both indirectly, as in the EBCTCG meta-analysis, and directly in one large, randomized trial suggest intravenous zoledronate, daily oral ibandronate and daily oral clodronate are of similar efficacy [85]. Most guidelines recommend intravenous zoledronate 4 mg started as soon as possible during (neo)adjuvant chemotherapy and continued thereafter every 6 months for a duration of at least 3 years. The intensive dosing schedules of zoledronate used in some of the clinical trials are not felt to be necessary and are associated with a much higher incidence of MRONJ. Alternatively, the patient may choose between daily oral clodronate 1,600 mg daily or oral ibandronate 50 mg daily as an alternative to intravenous therapy.

### 3.8 Disease recurrence prevention – Adjuvant denosumab

Although multiple studies have confirmed the efficacy of denosumab in preserving bone health, denosumab cannot be recommended for the prevention of disease recurrence in postmenopausal women with breast cancer [55,86].

Results of the Austrian Breast and Colorectal Cancer Study Group (ABCSG)-18 study of 3,420 postmenopausal women with hormone-sensitive early breast cancer who were receiving AI treatment suggested a DFS benefit of denosumab of 2.1 % at five years compared with placebo and a reported HR for DFS of 0.83 with a median follow-up of eight years [87,88]. However, no significant effects on breast cancer mortality were seen. The international, multicenter, Phase III D-CARE study, however, was unable to replicate these results, despite a study population at higher risk of recurrence than the ABCSG-18 study [89]. It randomized 4,509 women with high-risk early breast cancer to receive treatment with either denosumab or placebo the results of the D-CARE trial found that denosumab did not improve disease-related outcomes for this population of women.

The conflicting results between these two studies and the validity of comparing them are the subject of debate, but contributors to these opposing conclusions are likely to be differences study population, primary objectives, denosumab dose and dosing schedule [86].

Denosumab is not included as a recommended therapy in the American Society of Clinical Oncology–Ontario Health (Cancer Care Ontario) (ASCO-OH [CCO]) guidelines [55] or ESMO guidelines [12].

## 4 Conclusions and Future Directions

Maintaining bone health in women with early breast cancer remains an important management challenge in clinical practice, particularly with the increased use of extended adjuvant AI treatment. All women receiving AI treatment should be informed of this significantly increased risk and its consequences and have their individual fracture risk evaluated to determine an appropriate management strategy.

Regardless of fracture risk, preventative measures such as exercise and optimal calcium and vitamin D intake need to be ensured. If an increased fracture risk has been identified, suitable medical intervention should be proposed.

In terms of antiresorptive therapy, denosumab and bisphosphonates have both proven to be effective in this setting, although studies have shown that these agents have different effect sizes and safety/tolerability profiles, indicating that physicians and patients should aim to identify a personalized optimal treatment approach that best suits that individual.

Over and above their beneficial effects on bone health, adjuvant bisphosphonates, especially intravenous zoledronate, have been shown to significantly reduce bone recurrence and breast cancer mortality in postmenopausal women, and are now recommended in oncology treatment guidelines.

This updated position statement reflects the latest combined thinking on fracture and risk assessment in postmenopausal women with breast cancer who are receiving adjuvant AI therapy and summarizes the most suitable treatment modalities and an ideal treatment algorithm for the management of AIBL that both osteoporosis specialists and oncologists can adopt in clinical practice.

## CRediT authorship contribution statement

**Peyman Hadji:** Writing – review & editing, Writing – original draft, Conceptualization. **Matty Aapro:** Writing – review & editing. **Nasser Al-Dagri:** Writing – review & editing. **Majed Alokail:** Writing – review & editing. **Emmanuel Biver:** Writing – review & editing. **Jean-Jacques Body:** Writing – review & editing. **Maria Luisa Brandi:** Writing – review & editing. **Janet Brown:** Writing – review & editing. **Cyrille Confavreux:** Writing – review & editing. **Bernard Cortet:** Writing – review & editing. **Matthew Drake:** Writing – review & editing. **Peter Ebeling:** Writing – review & editing. **Erik Fink Eriksen:** Writing – review & editing. **Ghada El-Hajj Fuleihan:** Writing – review & editing. **Theresa A. Guise:** Writing – review & editing. **Nick C. Harvey:** Writing – review & editing. **Andreas Kurth:** Writing – review & editing. **Bente Langdahl:** Writing – review & editing. **Willem Lems:** Writing – review & editing. **Radmila Matijevic:** Writing – review & editing. **Eugene McCloskey:** Writing – review & editing. **Rossella Nappi:** Writing – review & editing. **Santiago Palacios:** Writing – review & editing. **Georg Pfeiler:** Writing – review & editing. **Jean-Yves Reginster:** Writing – review & editing. **René Rizzoli:** Writing – review & editing. **Daniele Santini:** Writing – review & editing, Conceptualization. **Sansin Tuzun:** Writing – review & editing. **Catherine Van Poznak:** Writing – review & editing. **Tobias De Villiers:** Writing – review & editing. **M. Carola Zillikens:** Writing – review & editing. **Robert Coleman:** Writing – original draft, Conceptualization.

## Declaration of competing interest

The authors declare that they have no known competing financial interests or personal relationships that could have appeared to influence the work reported in this paper.
